# Evolution of structural abnormalities in the rat brain following *in utero* exposure to maternal immune activation: A longitudinal *in vivo* MRI study

**DOI:** 10.1016/j.bbi.2016.12.008

**Published:** 2017-07

**Authors:** William R. Crum, Stephen J. Sawiak, Winfred Chege, Jonathan D. Cooper, Steven C.R. Williams, Anthony C. Vernon

**Affiliations:** a Department of Neuroimaging Institute of Psychiatry, Psychology and Neuroscience, King’s College London, De Crespigny Park, London SE5 8AF, UK; b Wolfson Brain Imaging Centre, Department of Clinical Neurosciences, University of Cambridge, Addenbrooke’s Hospital, Hills Road, Cambridge, UK; c Department of Psychosis Studies, Institute of Psychiatry, Psychology and Neuroscience, King’s College London, De Crespigny Park, London SE5 8AF, UK; d Department of Basic and Clinical Neuroscience, Institute of Psychiatry, Psychology and Neuroscience, King’s College London, Maurice Wohl Clinical Neuroscience Institute, 5 Cutcombe Road, London SE5 9RT, UK; e MRC Centre for Neurodevelopmental Disorders, King's College London, London SE1 1UL, UK

**Keywords:** Maternal immune activation, Poly(I:C), Magnetic resonance imaging, Volume, Cortex, schizophrenia

## Abstract

Genetic and environmental risk factors for psychiatric disorders are suggested to disrupt the trajectory of brain maturation during adolescence, leading to the development of psychopathology in adulthood. Rodent models are powerful tools to dissect the specific effects of such risk factors on brain maturational profiles, particularly when combined with Magnetic Resonance Imaging (MRI; clinically comparable technology). We therefore investigated the effect of maternal immune activation (MIA), an epidemiological risk factor for adult-onset psychiatric disorders, on rat brain maturation using atlas and tensor-based morphometry analysis of longitudinal *in vivo* MR images. Exposure to MIA resulted in decreases in the volume of several cortical regions, the hippocampus, amygdala, striatum, nucleus accumbens and unexpectedly, the lateral ventricles, relative to controls. In contrast, the volumes of the thalamus, ventral mesencephalon, brain stem and major white matter tracts were larger, relative to controls. These volumetric changes were maximal between post-natal day 50 and 100 with no differences between the groups thereafter. These data are consistent with and extend prior studies of brain structure in MIA-exposed rodents. Apart from the ventricular findings, these data have robust face validity to clinical imaging findings reported in studies of individuals at high clinical risk for a psychiatric disorder. Further work is now required to address the relationship of these MRI changes to behavioral dysfunction and to establish thier cellular correlates.

## Highlights

• Maternal immune activation leads to brain structural changes.
• Cortical volume decreases between post-natal day 50 and 100 and is static thereafter.
• Hippocampus volume decreases early and is maintained into adulthood.
• TBM reveals previously unappreciated changes in grey and white matter.

## 1 Introduction

Longitudinal magnetic resonance imaging (MRI) studies of typically developing individuals show that adolescence and early adulthood are dynamic and critical periods of brain maturation (Shaw et al., 2008, Sussman et al., 2016, Vijayakumar et al., 2016, Whitaker et al., 2016, Zhou et al., 2015, Sowell et al., 2001, Sowell et al., 2003). The disruption of this process by either genetic or environmental risk factors is therefore a potential susceptibility mechanism for the development of psychopathology in adult life, including schizophrenia (Millan et al., 2016, Insel, 2010, Rapoport et al., 2012). This is supported by data from longitudinal MRI studies of youth at high risk for psychosis (Cannon et al., 2015), youth with sub-threshold psychosis spectrum (PS) symptoms (Satterthwaite et al., 2016) and childhood onset-schizophrenia (COS) (Alexander-Bloch et al., 2014). These have established that structural and functional brain abnormalities similar to those observed in adult patients are already present early in life. Whether these are progressive (reflecting an on-going pathophysiological process) or static (reflecting early neurodevelopmental damage that arrests early in development) is controversial (Zipursky et al., 2013). Furthermore, the mechanisms driving these abnormalities remain unclear since MRI cannot currently visualise changes at the cellular level.

Whilst animal models cannot recapitulate the full phenotypic spectrum of psychiatric disorders, the presence or absence of developmental alterations in brain structure may be assessed in rodents with manipulations of either environmental or genetic risk factors for psychiatric disorders (Richetto et al., 2016, Hamburg et al., 2016). This can be informative for linking environmental or genetic disturbances with abnormalities of postnatal brain maturation and behaviour and mapping their cellular and molecular correlates (Piontkewitz et al., 2012a, Vernon et al., 2015, Hamburg et al., 2016, Richetto et al., 2016). Accordingly, cross-sectional MRI studies provide evidence for subtle, but enduring, brain structural abnormalities in the adult rodent brain following prenatal exposure to maternal immune activation (MIA) induced by polyriboinosinic-polyribocytidylic acid (POL) (Fatemi et al., 2008, Li et al., 2009, Li et al., 2010, Richetto et al., 2016, Piontkewitz et al., 2011b, Piontkewitz et al., 2009). To date, only a single longitudinal *in vivo* MRI study has been performed to assess the trajectory of these changes from adolescence to adulthood (Piontkewitz et al., 2011a). This study reported specific developmental trajectories of brain volumetric changes in both control and POL offspring that were region-, age-, and sex-specific (Piontkewitz et al., 2011a). Overall, POL offspring had smaller absolute volumes of the hippocampus, striatum and prefrontal cortex, and larger ventricular volume (Piontkewitz et al., 2011a). These data suggest prenatal exposure to POL leads to an abnormal postnatal trajectory of rat brain maturation and the regions affected are consistent with those identified from a prospective meta-analyses of brain volume abnormalities in patients with schizophrenia (van Erp et al., 2016).

However, recent data suggests that the rat brain continues to mature until PND180 (six months of age), before reaching a steady-state (Mengler et al., 2014). It is therefore unclear if brain volume abnormalities in POL-exposed rats continue to progress, remain static, or normalise with increasing post-natal age. Recent advances in image registration and computational analysis of rodent MRI data now permit analysis of such datasets in a brain-wide, operator-independent, voxel-wise fashion in a manner analogous to standard human structural MRI analysis pipelines (Lau et al., 2008, Lerch et al., 2008, Vernon et al., 2014). Whilst there are examples of such automated analysis in MIA models in the literature (Li et al., 2010, Richetto et al., 2016), these are cross-sectional, not longitudinal. Our laboratory has previously acquired T_2_-weighted structural MR images from the male offspring of rat dams exposed to either saline (SAL) or POL (4 mg/kg i.v.; GD15) at PND50, 100 and 180 as part of a study examining the trajectory of prefrontal cortex metabolites using ^1^H-MRS (Vernon et al., 2015). In the current study we set out to address the aforementioned issues by analysing this archival dataset using a combination of semi-automated atlas-based segmentation and longitudinal voxel-wise analysis using tensor-based morphometry (TBM).

## 2 Materials and methods

### 2.1 Animals

Animals were treated in accordance with the guidelines approved by the Home Office Animals (Scientific procedures) Act, UK, 1986 and European Union Directive 2010/63/EU. All animal experiments were given ethical approval by the ethics committee of King’s College London (United Kingdom). Eleven male and eleven female Sprague-Dawley rats (Charles River Laboratories, UK, 3 months of age) were used for timed mated breeding. Dams were housed individually under standard laboratory conditions in a temperature- (22 ± 2 °C) and humidity- (55 ± 10%) controlled room on a 12 h light–dark cycle (lights on at 6:00 am) with standard food and water available *ad libitum*.

### 2.2 Maternal immune activation (MIA)

This study utilises archival MRI data from a prior cohort of SAL and POL-exposed offspring, reported elsewhere (Vernon et al., 2015). No new animals were generated for this study. Time-mated breeding and induction of MIA were performed at Charles River Laboratories, UK, as previously reported (Vernon et al., 2015). Briefly, pregnant rats received either 4-mg/kg POL (*n* = 8; P9582, potassium salt; Sigma–Aldrich, UK) or 0.9% pyrogen-free SAL (*n* = 3) on gestational day (GD) 15. The POL was freshly prepared on the day of administration, dissolved in sterile pyrogen-free 0.9% saline to a final concentration of 50 mg/ml and administered intravenously (i.v. 0.1 ml per 100 g body weight) through the tail vein under mild physical constraint. The dose of POL was based on the pure concentration, which is 10% of the potassium salt. Immediately after injection animals were returned to their home cages. Maternal weight was recorded before and 24–48 h after the injection. Gestation length, litter size and offspring body weight were monitored in each group. After birth, pups were sexed and female pups culled on postnatal day (PND) 5. On PND21, male pups were weaned and housed 2–4 per cage with their littermates. On PND28, all of the SAL (*n* = 23 male pups from *n* = 3 independent litters) and POL (*n* = 59 male pups from *n* = 8 independent litters) rats were shipped to King’s College London and housed in the Biological Services Unit (BSU) as described (see Section 2.1).

The gestational stage for POL exposure (GD15) was selected based on previously validated MIA protocols from six independent laboratories using rats (Mattei et al., 2014, van den Eynde et al., 2014, Yee et al., 2012, Zuckerman et al., 2003, Dickerson et al., 2010, Ballendine et al., 2015). In C57/Bl6 mice, differential phenotypes emerge following MIA if the insult is performed either early (GD9) or late (GD17) in gestation (Meyer, 2014, Bitanihirwe et al., 2010, Meyer et al., 2006, Meyer et al., 2008). A recent report suggests that GD10 and GD19 in the rat are also neurodevelopmental stages that are sensitive to MIA, resulting in PPI and working memory dysfunction, respectively (Meehan et al., 2016). However, the ‘spectrum’ of schizophrenia-relevant brain and behavioral changes reported after MIA exposure at GD14-15, were not observed (Meehan et al., 2016). Those time-points may not therefore be as sensitive a window for MIA as GD15. We therefore considered GD15 to be a rational start point for investigations of neuroimaging abnormalities following POL exposure.

Following shipping to KCL, pups were left undisturbed until PND45, when they were weighed and allocated at random into experimental groups for study. The data presented in this manuscript are based on longitudinal *in vivo* T_2_-weighted structural MRI (sMRI) scans acquired in the same session as ^1^H-MRS data, which we reported previously (Vernon et al., 2015). However, due to time constraints, structural MRI data were only acquired from  *n* = 6 POL litters. No more than two animals were selected from each POL litter and no more than four from each SAL litter (Vernon et al., 2015). The remaining animals were utilised for additional experiments to be reported elsewhere.

### 2.3 Structural MRI acquisition

A 7T small-bore horizontal magnet MRI scanner (Agilent Technologies Inc. Santa Clara, USA) equipped with a custom-made quadrature volume radiofrequency (RF) coil (43 mm inner diameter, Magnetic Resonance Laboratory, Oxford) was used for all MR image acquisition (Vernon et al., 2015). Briefly, animals were anaesthetized throughout scanning using 1.0% isoflurane in a mixture of medical air: oxygen (70:30) delivered at 1 L/min. Body temperature (regulated at 37 °C), blood oxygen saturation and respiration rate were monitored for the duration of the scan(s). T_2_-weighted MR images were acquired using a 2D Fast Spin Echo (FSE) sequence: repetition time (TR)/effective echo time (TE) = 4000/60 ms, averages = 8, field of view = 30 × 30 mm, matrix size 128 × 128, (in-plane resolution 234 μm) with 45 contiguous coronal slices, 0.6 mm thick (Vernon et al., 2012).

### 2.4 Semi-automated atlas-based segmentation analysis of MR images

Analysis of total and regional brain volumes were performed using a semi-automated atlas-based segmentation approach using the SPM mouse toolbox (http://www.spmmouse.org) implemented in the Statistical Parametric Mapping (SPM) 8 software package (Wellcome Department of Clinical Neurology, London; http://www.fil.ion.ucl.ac.uk) (Sawiak et al., 2009). A mean image of the entire dataset (*n* = 60 scans) was made using an iterative registration procedure to provide a population specific template (PST; Supplementary Fig. 1). Total brain volumes were derived using the “get totals” function in SPM8. The PST was then parcellated into five regions of interest (ROI) in the left and right hemispheres for (a) the anterior cingulate cortex (ACC), (b) corpus striatum (STR), (c) lateral ventricles (LV), (d) dorsal hippocampus (dHPC) and (e) ventral hippocampus (vHPC; Supplementary Fig. 2) using ITK-snap (http://www.itksnap.org) (Yushkevich et al., 2006). These ROI were chosen *a priori* on the basis of their prior investigation in this model (Piontkewitz et al., 2011a) and their central involvement in several human psychiatric disorders with a putative neurodevelopmental origin, including schizophrenia (Haijma et al., 2013, van Erp et al., 2016). ROI delineations were performed using established criteria for neuroanatomical segmentation of rat brain MR images (Piontkewitz et al., 2011a, Vernon et al., 2011b, Vernon et al., 2011a, Vernon et al., 2012, Vernon et al., 2010, Harrison et al., 2015). Individual MR images from SAL and POL exposed offspring at each time-point were transformed to this atlas space using affine registration and assigned a grey matter (GM) probability distribution modulated by the Jacobian determinant of the transformation. Using a segmentation-propagation approach (Norris et al., 2013) the ROI masks for each structure were propagated from the PST into the native space of each individual rat MR image, using the inverse of the deformation parameters obtained whilst spatially normalizing the images. This provides the spatial correspondence between every voxel in the average image and their corresponding positions in each single rat brain image. Following segmentation-propagation, for quality control purposes, all individual MR images were visually inspected to ensure anatomical labels were accurately positioned. No data were excluded on this basis.

### 2.5 Statistical analysis of atlas-based segmentation data

A key conclusion from prior MR imaging studies of rodents is that whilst anatomical variability is low (∼5%), this remains the single most significant source of variance in imaging studies (Lerch et al., 2012). This variability largely derives from inter-animal variation in the total brain volume, rather than specifically that of local structures (Lerch et al., 2012). Furthermore, there are tight correlations between volumes of some structures and total brain volumes, particularly for the hippocampus (Lerch et al., 2012). Prior MRI analyses of the POL rat model have not accounted for this variable (Piontkewitz et al., 2011a, Piontkewitz et al., 2011b, Piontkewitz et al., 2009). To address this, the volumes of each brain region derived from the atlas-based segmentation were analysed as absolute values, but also relative values after normalisation to total brain volume from the same animal. Data from the left and right hemispheres were summed together. Because of the low number of control litters, atlas-derived volumes were compared using the number of litters (i.e. mothers) instead of offspring, in the statistical analysis, as described previously (Garbett et al., 2012, Vernon et al., 2015). The volume data from each individual rat from a given litter is averaged to give a mean value for that particular litter. We therefore proceeded to compare data between SAL (*n* = 3) and POL (*n* = 6)-exposed litters using a 2-way repeated measures (RM) ANOVA with one between subject-factor (MIA) and one within-subject factor (time) followed by *post-hoc* Bonferroni evaluation of any significant MIA × Age interactions. All statistical analyses were carried out using SPSS® 21.0 software (SPSS Inc. IBM, NY, USA) with α-level of 0.05.

### 2.6 Longitudinal tensor based morphometry (TBM)

An operator-independent whole-brain comparison of SAL and POL litters at each imaging time-point was then performed using an automated image processing pipeline (Crum et al., 2013a), which has proven robust in rodent imaging applications (Harrison et al., 2015, Vernon et al., 2014). A single brain from the PND100 time-point was chosen as a canonical reference and manually aligned with standard coordinate axes. Masks that (a) fitted tightly around the canonical brain and (b) included a boundary region outside the canonical brain were then defined manually for analysis and registration respectively. All scans were registered to this reference with 9 degrees of freedom (dof) (i.e. rigid-body translation and rotation in 3D together with correction for global scaling differences across the cohorts) using a previously published method (Jenkinson et al., 2002) based on FLIRT (Crum et al., 2013b). To measure serial volume changes within group, across adjacent time-points, further 9dof registrations were performed for the PND100 scan to the corresponding PND50 scan, and each PND180 scan to the corresponding PND100 scan for each animal in each group. These fluid registration steps result in a dense displacement field that maps each point in the original scan to the corresponding point on the reference mean. From this map, an estimate of apparent volume difference (the Jacobian determinant, *J*) between the scan and the population mean at each voxel can be obtained. TBM analysis then applies voxel-wise non-parametric t-tests to these volume difference estimates to determine the location of statistically significant differences in brain tissue volume of SAL compared with POL. Collectively, these analyses allow for the comparison of differences in volume within each treatment group (SAL or POL) at each time point (PND50 – 100 and 100 to 180). These maps thus show effects of age and MIA together. To determine the specific differences in local structural changes, between groups, across time, additional high-dimensional non-rigid registrations (Crum et al., 2005) were performed between each pair of serial scans (i.e. PND100 to PND50, and PND180 to PND100). The resulting maps show the difference in volume changes (ΔJ) across the whole brain, between the two groups (SAL and POL), across a fixed period of time (either PND50 to 100 or 100 to 180). Significance levels were corrected for multiple comparisons across voxels using the false discovery rate (FDR) (Genovese et al., 2002), based on simulations of recoverable atrophy in the mouse brain and number of true positive and false positive voxels recovered from TBM analysis (van Eede et al., 2013).

## 3 Results

### 3.1 Longitudinal time course of absolute brain volume changes following pre-natal POL exposure

**Fig. 1:**
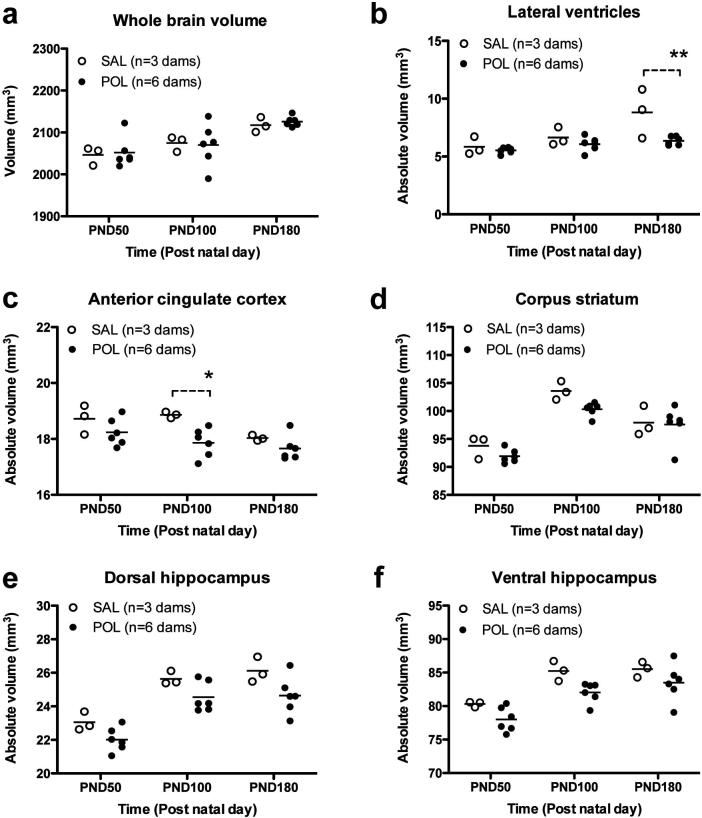
Prenatal exposure to POL on GD15 results in changes in the absolute volumes of key brain structures derived using atlas-based segmentation. (a) Prenatal POL treatment did not affect total brain volume, but led to a reduction in the absolute volume (not corrected for total brain volume) of (b) the lateral ventricles by PND180. The absolute volume of (c) the anterior cingulate cortex was also reduced, but there was no effect on (d) corpus striatum volume. Prenatal POL treatment also lead to a reduction in the absolute volumes of (d) the dorsal hippocampus and (e) ventral hippocampus. Data shown are litter means from n = 3 SAL dams and n = 6 POL dams. ^*^p < 0.05; ^**^p < 0.01; SAL vs. POL using *post-hoc* Bonferroni correction for multiple comparisons based on a significant age × MIA interaction.

Total brain volume increased with age at each post-natal time-point, but did so comparably between SAL and POL-exposed litters (Fig. 1a; Table 1). We then compared the effects of MIA on absolute volumes of the *a priori* ROIs. LV absolute volumes increased with age in both groups of litters (Fig. 1b). ANOVA yielded significant main effects of age, MIA and age × MIA interaction (Table 1). *Post-hoc* testing of the interaction confirmed significantly smaller absolute LV volume in POL litters compared to SAL at PND180 (Table 1; Fig. 1b). Similarly, absolute ACC volume decreased with age in both groups of litters (Fig. 1c). ANOVA yielded significant main effects of age, MIA and age × MIA interaction (Table 1). *Post-hoc* testing of the interaction confirmed a significantly smaller absolute ACC volume in POL litters compared to SAL at PND90 (Table 1; Fig. 1c).

**Table 1:** Two-way repeated measures ANOVA statistics for atlas based segmentation analysis using absolute volumes for each brain region measured. Maternal immune activation (MIA, [POL]) treatment served as between-subject factor and time as within-subject factor. ^a^*Post-hoc* tests were only performed for significant age × MIA interactions using Bonferroni’s *post-hoc* test corrected for multiple comparisons. ANOVA, analysis of variance; MIA, maternal immune activation n.s., not significant.

Brain region	Two way repeated measures ANOVA			*Post-hoc* test (Bonferroni’s test for multiple comparisons)^a^
	Within subjects		Between groups	
	Age	Age × MIA interaction	MIA	
Whole brain	F(2,7) = 13.87; *p* < 0.001	F(2,7) = 0.12; n.s.	F(1,7) = 0.04; n.s.	None performed
Lateral ventricles	F(2,7) = 14.84; *p* < 0.001	F(2,7) = 6.09; *p* < 0.05	F(1,7) = 6.56; *p* < 0.05	P180 SAL vs. P180 POL; *p* < 0.01
Anterior cingulate cortex	F(2,7) = 15.43; *p* < 0.001	F(2,7) = 3.78; *p* < 0.05	F(1,7) = 5.00; n.s.	P90 SAL vs. P90 POL; *p* < 0.05
Corpus striatum	F(2,7) = 44.72; *p* < 0.001	F(2,7) = 1.16; n.s.	F(1,7) = 3.07; n.s.	None performed
Dorsal hippocampus	F(2,7) = 19.64; *p* < 0.001	F(2,7) = 0.18; n.s.	F(1,7) = 10.52; *p* < 0.05	None performed
Ventral hippocampus	F(2,7) = 28.13; *p* < 0.001	F(2,7) = 0.33; n.s.	F(1,7) = 6.53; *p* < 0.05	None performed

The absolute STR volume showed an inverted U-shaped trajectory, increasing between PND50 and 100 and decreasing thereafter between PND100 to 180. This was comparable between SAL and POL litters, with ANOVA yielding a significant main effect of age, but not MIA or age × MIA interaction (Table 1 and Fig. 1d). The absolute dHPC and vHPC volumes increased with age in both groups of litters (Fig. 1e, f). ANOVA yielded significant main effects of age and MIA, but no age × MIA interaction (Table 1; Figs. 1e, f). Indeed, the hippocampus volumes are clearly reduced in POL as compared to SAL litters at all time-points (Fig. 1e, f).

### 3.2 Longitudinal time course of relative brain volume changes following pre-natal POL exposure

**Fig. 2:**
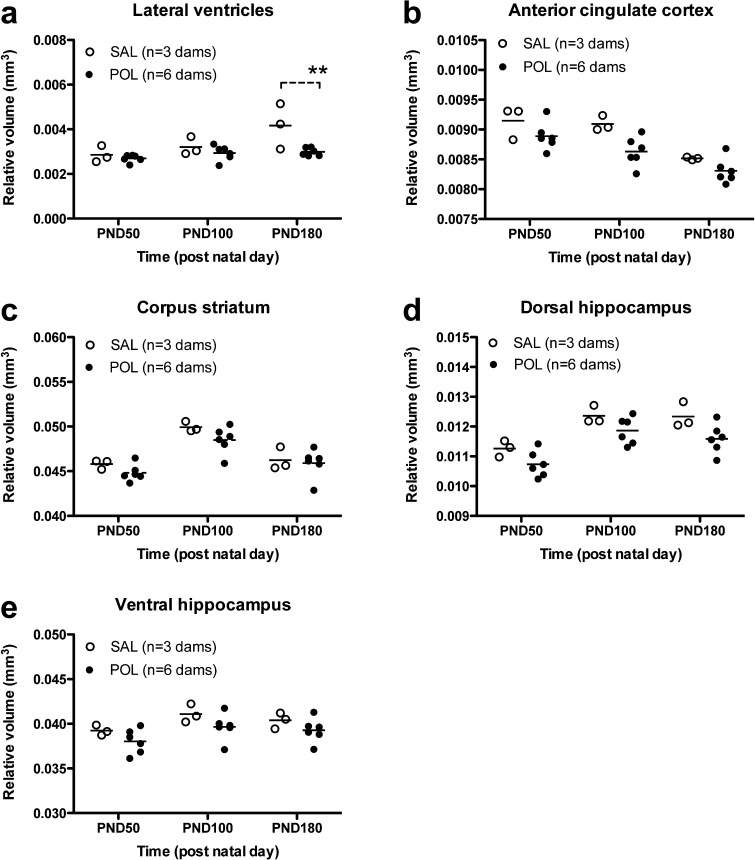
Prenatal POL treatment led to a reduction in the relative volume (corrected for total brain volume) of (a) the lateral ventricles at PND180 and (b) ACC, with no effect on (c) corpus striatum volume. Prenatal POL treatment also leads to a reduction in the relative volumes of (d) the dorsal hippocampus and (e) ventral hippocampus. Data shown are litter means from n = 3 SAL dams and n = 6 POL dams. ^**^p < 0.01; SAL vs. POL using *post-hoc* Bonferroni correction for multiple comparisons based on a significant age × MIA interaction.

Brain structure volumes can be normalised to total brain volume to correct for inter-animal variation in brain size (Lerch et al., 2012). We therefore re-analysed the volume data derived from the atlas-based segmentation approach after normalization to total brain volume for each individual animal in each litter. The relative LV volume showed identical trends to the absolute LV volume data and increased with age in both groups of litters. ANOVA again yielded significant main effects of age, MIA and age × MIA interaction (Table 2). *Post-hoc* testing of the interaction confirmed that relative LV volume is smaller in POL litters at PND180 as compared to SAL litters (Table 2; Fig. 2a). In contrast, whilst the relative ACC volume declined with age in both groups of litters, ANOVA yielded only significant main effects of age and MIA, but no age x MIA interaction (Table 2, Fig. 2b). The data for the relative volumes of the STR, dHPC and vHPC were also similar to the trends in the absolute volume data for these regions (Table 2 and Figs. 2c, d, e), with one exception. The ANOVA did not yield a significant main effect of MIA for the relative volume of the vHPC (Table 2). However, this may simply reflect the low power of this dataset, particularly for control SAL litters (*n* = 3), rather than a genuine regional difference in effects of MIA on hippocampus volume *per se*. Indeed, closer inspection of Fig. 2d and e clearly shows however that the relative volume of the dHPC and vHPC is reduced in the POL litters as compared to the SAL litters at all time-points.

**Table 2:** Two-way repeated measures ANOVA statistics for atlas based segmentation analysis using relative (i.e. normalised to total brain volume) volumes for each brain region measured. Maternal immune activation (MIA, [POL]) treatment served as between-subject factor and time as within-subject factor. *^a^Post-hoc* tests were only performed for significant age × MIA interactions using Bonferroni’s *post-hoc* test corrected for multiple comparisons. ANOVA, analysis of variance; MIA, maternal immune activation n.s., not significant.

Brain region	Two way repeated measures ANOVA			*Post-hoc* test (Bonferroni’s test for multiple comparisons)^a^
	Within subjects		Between groups	
	Age	Age × MIA interaction	MIA	
Lateral ventricles	F(2,7) = 12.74; *p* < 0.001	F(2,7) = 6.03; *p* < 0.05	F(1,7) = 5.77; *p* < 0.05	P180 SAL vs. P180 POL; *p* < 0.01
Anterior cingulate cortex	F(2,7) = 26.44; *p* < 0.001	F(2,7) = 1.18; n.s.	F(1,7) = 7.36; *p* < 0.05	None performed
Corpus striatum	F(2,7) = 38.29; *p* < 0.001	F(2,7) = 0.69; n.s.	F(1,7) = 1.76; n.s.	None performed
Dorsal hippocampus	F(2,7) = 24.93; *p* < 0.001	F(2,7) = 0.31; n.s.	F(1,7) = 6.52; *p* < 0.05	None performed
Ventral hippocampus	F(2,7) = 6.69; *p* < 0.01	F(2,7) = 0.06; n.s.	F(1,7) = 3.10; n.s.	None performed

### 3.3 TBM analysis complements atlas-based segmentation and reveals additional differences between groups not seen with atlas-based segmentation

**Fig. 3:**
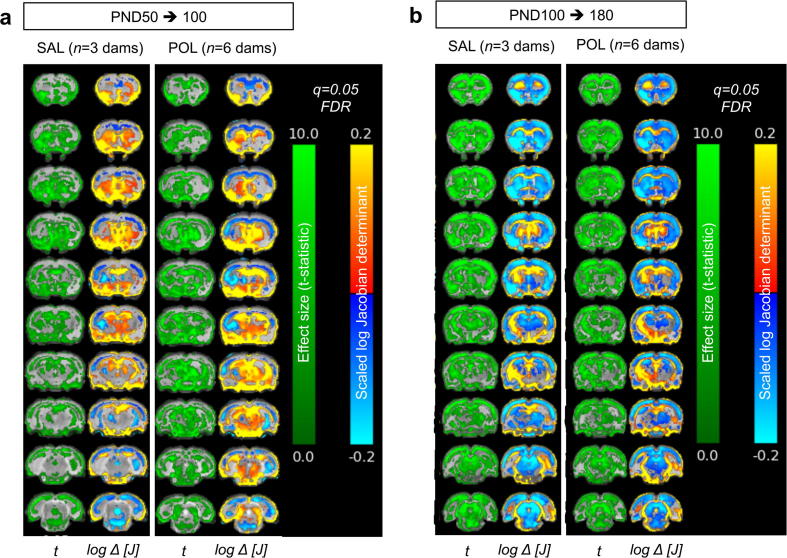
Longitudinal tensor based morphometry analysis of structural brain abnormalities within SAL and POL litters between (a) PND50 and PND100 and (b) PND100 to 180. Both the effect size (*t*; t-statistic) and relative change in the log scaled jacobian determinant (log ΔJ) are shown. Hot colours (red-yellow) indicate volumetric expansions, whilst cold colours (blue-cyan) indicate volumetric contractions. Data are corrected for multiple comparisons using the False Discovery Rate (FDR) at q = 0.05. (For interpretation of the references to colour in this figure legend, the reader is referred to the web version of this article.)

**Fig. 4:**
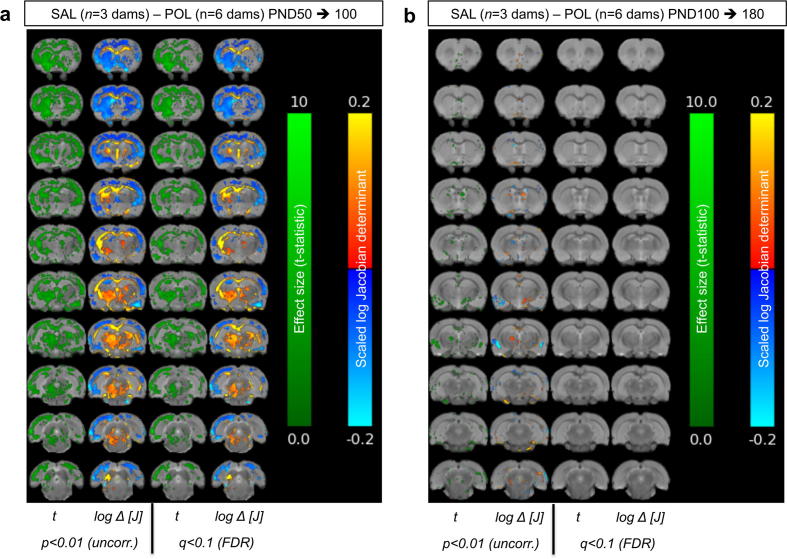
Longitudinal tensor based morphometry analysis of structural brain abnormalities between SAL and POL litters from (a) PND50 to 100 and (b) PND100 to PND180. Both the effect size (*t*; t-statistic) and relative change in the log scaled jacobian determinant (log ΔJ) are shown. Hot colours (red-yellow) indicate volumetric expansions, whilst cold colours (blue-cyan) indicate volumetric contractions. Data are corrected for multiple comparisons using the False Discovery Rate (FDR) at q = 0.10 and at trend-level (p < 0.01 uncorrected). (For interpretation of the references to colour in this figure legend, the reader is referred to the web version of this article.)

TBM was used to compare SAL and POL brains at the three time points scanned (Fig. 3, Fig. 4). Between PND50 and 100 (Fig. 3a), within each group, the volumes of the prefrontal, motor, somatosensory, auditory and visual cortex, dorsal thalamic nuclei, ventral midbrain and brain stem decrease significantly (q = 0.05; Fig. 3a). In contrast, ventricular, striatal, hippocampal, ventral thalamic and white matter volumes increase significantly (q = 0.05; Fig. 3a). Qualitatively, these volumetric decreases were stronger in the frontal cortex, ventral thalamic nuclei and ventral midbrain of POL litters relative to SAL controls (Fig. 3a). In the second time-window (PND100 – 180), within both groups, the cortex, midbrain and brain stem show continued significant volume decreases, with most of the cortex now affected (q = 0.05; Fig. 3b). White matter volumes continue to significantly increase, whilst thalamic and striatal volumes significantly decrease and hippocampus volume remains stable (q = 0.05; Fig. 3b). However, qualitatively comparing SAL and POL litters, specific effects due to POL exposure are very difficult to discern from these maps (Fig. 3b).

In order to quantitatively visualise specific volumetric differences between the groups in each time-window, additional high-dimensional non-rigid registrations were performed between each pair of serial scans (i.e. PND100 to PND50, and PND180 to PND100; Fig. 4a, b). These data confirm that between PND50 to 100, there is a significantly larger decrease in the volume of frontal, motor, somatosensory, parietal, visual and auditory cortices, the striatum, nucleus accumbens and amygdala in POL litters as compared to SAL (q = 0.1; Fig. 4a). In contrast, the volume increase in the corpus callosum and external capsule as well as the ventral thalamus and midbrain is significantly greater in POL litters as compared to SAL controls (q = 0.1; Fig. 4a). Between PND100 – 180, only sparse clusters of voxel show volumetric changes and only at a trend level (*p* < 0.01 uncorrected; Fig. 4b). For example, the POL litters show greater decrease in ventricular and amygdala volumes, but these do not survive FDR correction (Fig. 4b).

## 4 Discussion

The brain morphological phenotype of the GD15 MIA rat model has been previously reported using histological techniques (Piontkewitz et al., 2012b) and manual morphometry from longitudinal MRI scans (Piontkewitz et al., 2011a). Here we looked for differences between rat brains exposed to either SAL or POL on GD15 with both a semi-automated (atlas-based segmentation) and fully automated (tensor based morphometry) technique, to reveal a complete picture of neuroanatomical changes in POL-exposed litters, including regions of expansion as well as atrophy. The principle findings from each of our analysis methods are discussed below.

Prior work in rats suggests that prenatal exposure to POL on GD15 does not change the overall shape of the maturational trajectories of key brain structures, but rather leads to maturation- and sex-dependent volumetric deviation, with volume reduction in the hippocampus, prefrontal cortex and striatum accompanied by ventricular hypertrophy as compared with controls (Piontkewitz et al., 2011a). This study utilised manual segmentation of regions-of-interest (ROIs). Whilst robust, this technique is labour-intensive and prone to intra- and inter-rater bias. In the current study we sought to replicate these data using a semi-automated atlas-based segmentation. Here, the brain ROIs are defined manually on a population specific template and using the inverse transformation of the native images to this template space, are propagated back onto the original scans and the volume calculated (Crum et al., 2016). This approach is not only faster, but the manual segmentation of brain structures is performed on an average MR image of, in this case, 60 rat brains, which factors out the occurrence of artifacts and positional differences as would be seen on individual brains (Dorr et al., 2008). Together, this increases the clarity of the image for improved structural boundary determination (Dorr et al., 2008).

Taking these factors into account, our atlas-based segmentation reveals that in both SAL and POL litters, the longitudinal volumetric changes spanning from PND50 to PND180 were region-specific. The ACC declined in volume between PND50 and 100, plateauing thereafter. This volume reduction is greater in POL offspring between PND50 and 100, but not thereafter, consistent with and extending prior work (Piontkewitz et al., 2011a). We replicated the inverted U-shaped trajectory of striatum volume, but POL exposure had no effect on this metric using our atlas-based approach, in contrast to prior work (Piontkewitz et al., 2011a). In both litters, dHPC, vHPC and LV volumes increased between PND50 to 100, but tended to plateau thereafter by PND180. Consistent with prior studies, hippocampus volume was reduced in POL litters (Piontkewitz et al., 2011a), but unexpectedly LV volumes were smaller. Overall, these results were largely unaffected by comparing either absolute or relative (i.e. normalised) volumes, with significant age × MIA interactions present in both absolute and relative volume datasets for the LV, but not other regions. These data suggest MIA affects the maturational trajectory of the ventricles, but the statistics do not allow a conclusion on changes in trajectory for the other brain regions measured. The finding of reduced LV volume is in stark contrast to prior findings of ventricular hypotrophy in this rat model (Piontkewitz et al., 2011a, Piontkewitz et al., 2011b, Piontkewitz et al., 2009) and in schizophrenia generally (Haijma et al., 2013, van Erp et al., 2016). Importantly, our control litter sample size was small, which may have affected these data, thus our findings should be interpreted cautiously. It is also plausible that this discrepancy reflects methodological differences between the studies. These could include a differential sensitivity of rat strains to MIA (Wistar vs. Sprague-Dawley), a differential POL administration protocol (use of isoflurane or not) or even a systematic bias inherent to the automated method. Prior work in the mouse brain shows that this bias becomes more evident as the size of the segmented structure decreases, with the greatest deviations observed in the lateral ventricles (Lau et al., 2008). Despite this, the manual and automated measurements correlated strongly in this dataset, justifying the use of reproducible, automated segmentation rather than manual approaches that suffer from intra-/inter-rater variability (Lau et al., 2008).

This *a priori* approach however negates one of the major benefits of MRI, which is the ability to image the entire brain in a reasonable amount of time. Limiting the analyses to a small number of ROIs also reduces the rich information available in MRI to a single composite number. To address this we present the first fully automated, brain-wide longitudinal TBM analysis, of a rat MIA model. In broad terms, TBM complements the atlas-based analysis. For example, the two methods find the same volume reductions in the ACC and TBM confirms this effect is greater in the POL litters as compared to SAL. Similarly, TBM also identifies a decrease in LV volume with increasing age in POL litters, arguing against this being the result of systematic bias in the segmentation protocol. TBM is also clearly more sensitive to subtle anatomical changes, detecting reduced striatal volume in the POL litters, which the atlas-based segmentation did not. Notably, differences in results between segmentation and voxel-wise approaches also exist in clinical imaging, including in schizophrenia (Giuliani et al., 2005). Differential sensitivities between these techniques are therefore not unexpected, since they provide different types of information, but this does not mean that either technique produces incorrect results (Sawiak et al., 2009). Manual morphometry, which we used to delineate the ROIs in our semi-automated analysis, depends largely on the skill of the operator to discriminate between and delineate, different structures on MR images and provides volumetric data from individual brains. TBM on the other hand is a quantitative image analysis technique, which evaluates information contained within the vector field generated by the nonlinear warping of individual MR images to a reference template (Lau et al., 2008). Given these differences in the two techniques, it should be expected that the results would not be in perfect accordance. Moreover, since we cover the whole brain, there is the potential to identify regions of previously unappreciated volume loss. For example our analysis shows that POL litters have volume decreases in several other cortical areas besides the ACC, as well as the nucleus accumbens and amygdala to name a few. There are also previously unappreciated increases in the volume of the thalamus and ventral midbrain and interestingly, the major white matter tracts in POL-exposed litters.

The data from both methods suggests that with the exception of the LV, the grey and white matter brain structural differences between SAL and POL-exposed litters were maximal between PND50 and 100 with no differences between the groups thereafter. This would suggest the effects of MIA on brain structure occur early in life, but are then static and do not show further progression. This finding is at least consistent with our recent data in a mouse MIA model, which in adult mice (12 weeks of age), there were relatively sparse volume differences between the MIA and control groups (Richetto et al., 2016). The work of others suggests these volume changes may occur during a critical window of brain maturation for example, adolescence, (Piontkewitz et al., 2011a) or even earlier in neurodevelopment. However, our current data cannot confirm either of these suggestions.

An important question is what is the cellular and molecular basis of these structural changes? Prior studies provide evidence that disruption of neurogenesis, vascular integrity, metabolic abnormalities and altered excitation – inhibition balance may be linked to hippocampus and or prefrontal cortex volume loss in following MIA (Hadar et al., 2015, Patrich et al., 2016, Piontkewitz et al., 2012b, Vernon et al., 2015, Meyer et al., 2008, Nyffeler et al., 2006, Richetto et al., 2014). More recently combining MRI and genome-wide transcription or proteomics analysis suggests MIA induces myelin dysfunction, which will be important to explore in terms of our observations of increased white matter volume (Farrelly et al., 2015, Richetto et al., 2016). There is also evidence for decreased levels of in synaptic proteins in the hippocampus and PFC of POL-exposed mice (Giovanoli et al., 2015, Giovanoli et al., 2016), whilst evidence for microglial activation is equivocal (Giovanoli et al., 2015, Giovanoli et al., 2016; Mattei et al., 2014, Eßlinger et al., 2016). Further work to link neuroimaging and neuropathology in this model is therefore required.

Although extrapolation from animal data to clinical disorders must be made with extreme caution, our data and those of others (Piontkewitz et al., 2011a) may well have relevance for adult-onset neuropsychiatric disorders in which neurodevelopmental factors are believed to play a role. For example, longitudinal structural neuroimaging studies in youth with psychosis spectrum (PS) symptoms, genetic and clinical high-risk individuals have described volumetric reductions or thinning of the frontal cortex, as well as volume decreases in temporal, thalamic and limbic brain regions that occur before, through and after transition to psychosis (Cannon et al., 2015, Dazzan et al., 2012, Lawrie et al., 2001, Rapoport et al., 2012, Satterthwaite et al., 2016, Sun et al., 2009, van Haren et al., 2011). Interestingly, recent data from youth with PS symptoms also reveals expanded white matter volumes in this population (Satterthwaite et al., 2016). However, our data do not recapitulate the consistent findings of ventricular hypertrophy observed in schizophrenia patients. A recent meta-analysis of ventricular volume in schizophrenia revealed that in several instances the differences were noted to be due to the ventricular size of the control samples (Sayo et al., 2012). Importantly, our control litter sample size was small, which may have affected these data, thus our LV findings should be interpreted cautiously.

## 5 Conclusions

The findings of the current study lend support to the suggestion that prenatal exposure to MIA leads to structural brain changes, which have face validity to human neuropsychiatric disorders of neurodevelopmental origin. Further work is required to validate this against behavioral and post-mortem phenotypes in this model.

## Funding sources

Funding from the Medical Research Council (GrantID: G0701748 and G1002198) whom we thank for their generous financial assistance supported this study. The MRC had no further role in study design; in the collection, analysis and interpretation of data; in the writing of the report; and in the decision to submit the paper for publication.
