# Supplementary material for: Evolution of structural abnormalities in the rat brain following in utero exposure to maternal immune activation: A longitudinal in vivo MRI study
Source: Brain Behav Immun. 2017 Jul;63:50–9. doi: 10.1016/j.bbi.2016.12.008 (PMC5441572; doi:10.1016/j.bbi.2016.12.008)
Supplement: Supplementary data 1 [file mmc1.docx]

**Supplementary information**

**
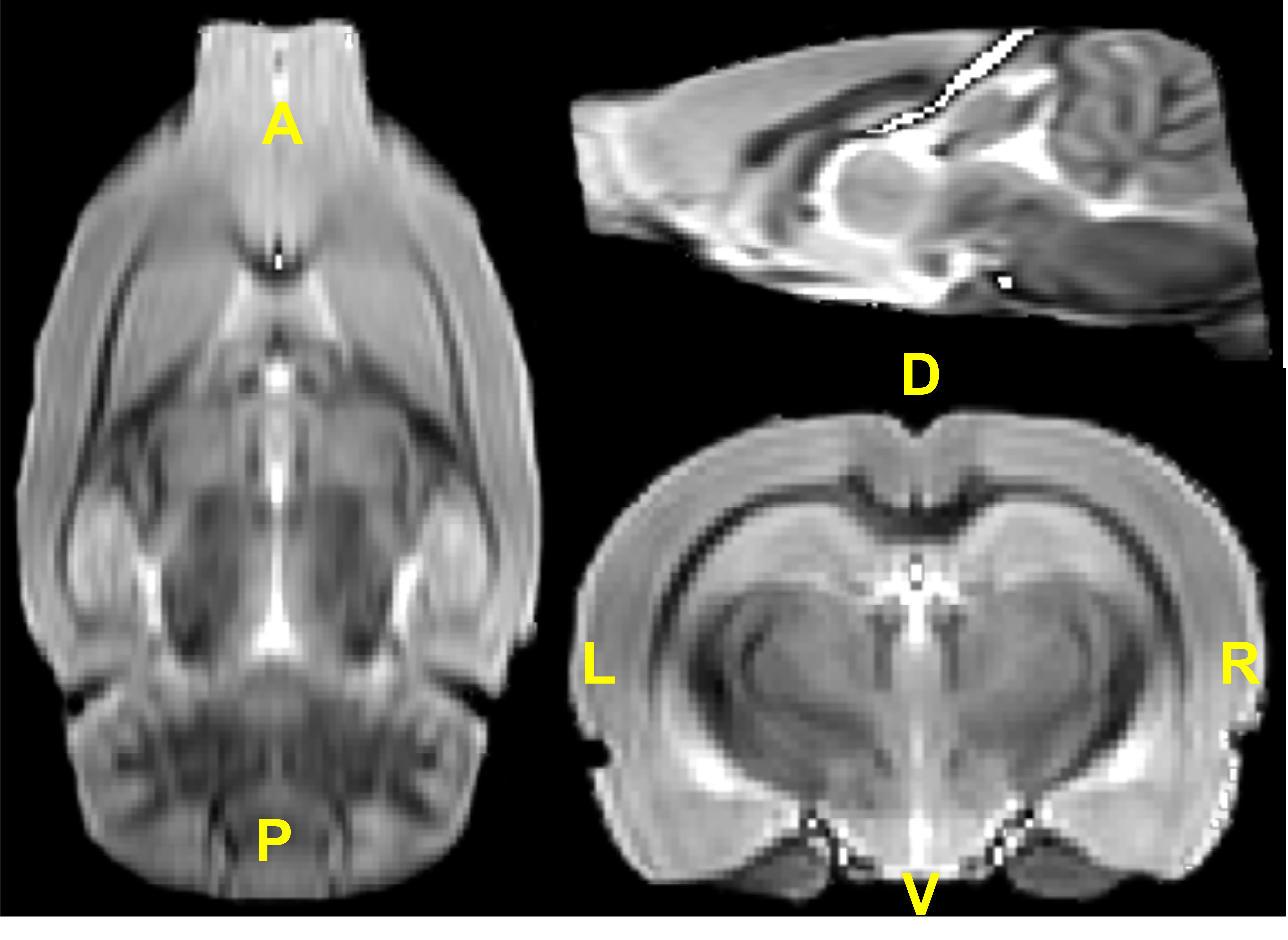
**

**Supplementary Figure 1.** Axial, sagittal and coronal views of the mean *T*_2_-weighted MR image of the entire dataset (*N*=60 scans) generated using an iterative registration procedure to provide a population specific template for atlas-based segmentation, A, anterior, P, posterior, D, dorsal; V, ventral; L, left and R, right.


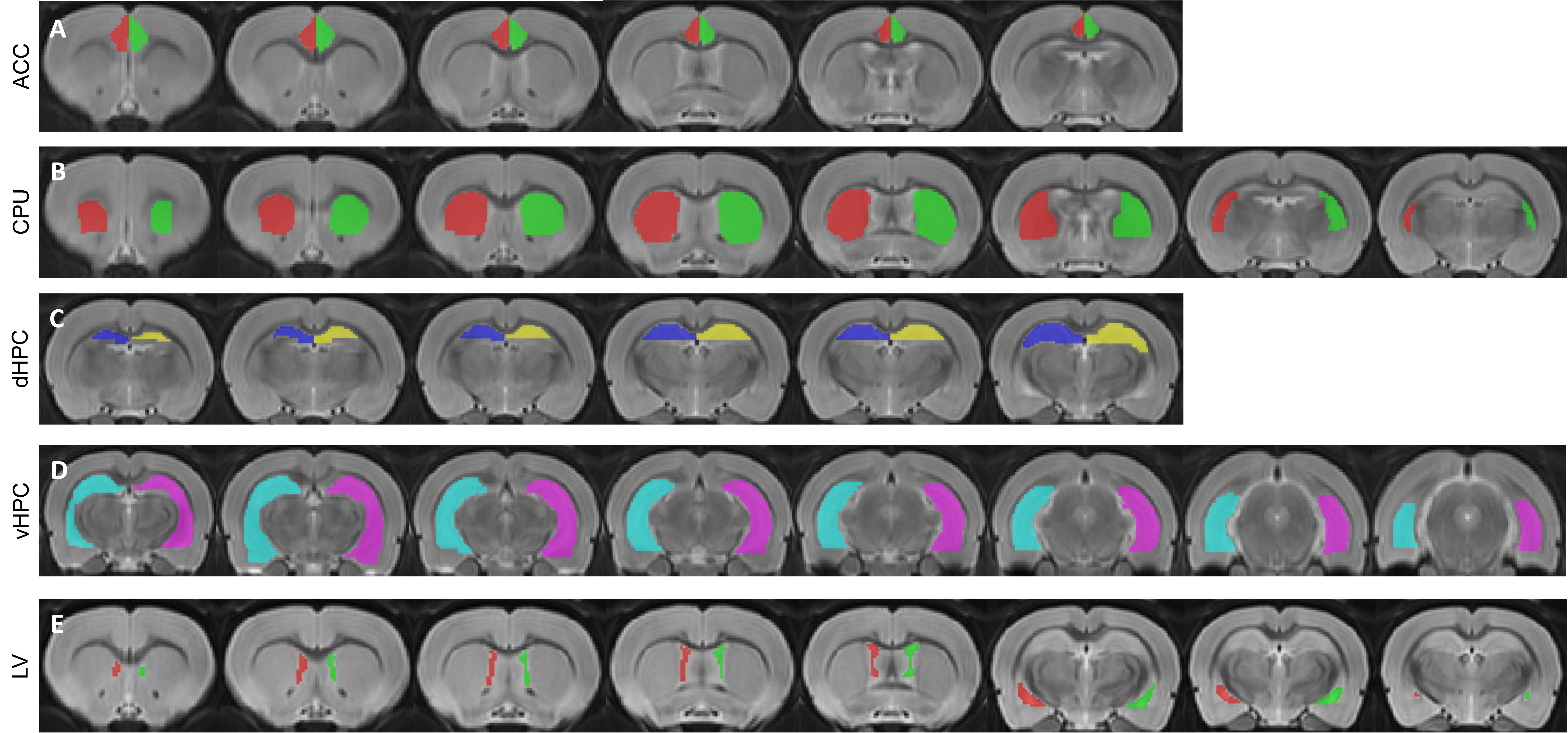


**Supplementary Figure 2.** Representative illustration of the region of interest (ROI) contours used for atlas-based segmentation, that were selected *a priori* on the basis of prior work and their relevance to neurodevelopmental psychiatric disorders. Contours were segmented for the left and right hemispheres separately on the population specific template shown in supplementary figure 1 by a single trained operator (ACV) with high fidelity (Intra-class correlation coefficient >0.95) using previously published anatomical criteria. **(A)** Anterior cingulate cortex (ACC), **(B)** Corpus striatum (CPU), **(C)** dorsal hippocampus (dHPC), **(D)** ventral hippocampus (vHPC) and **(E)** lateral ventricles.
